# The (In)Effectiveness of Simulated Blur for Depth Perception in Naturalistic Images

**DOI:** 10.1371/journal.pone.0140230

**Published:** 2015-10-08

**Authors:** Guido Maiello, Manuela Chessa, Fabio Solari, Peter J. Bex

**Affiliations:** 1 UCL Institute of Ophthalmology, University College London, London, United Kingdom; 2 Department of Informatics, Bioengineering, Robotics and System Engineering, University of Genoa, Genoa, Italy; 3 Department of Psychology, Northeastern University, Boston, MA, United States of America; University of Muenster, GERMANY

## Abstract

We examine depth perception in images of real scenes with naturalistic variation in pictorial depth cues, simulated dioptric blur and binocular disparity. Light field photographs of natural scenes were taken with a Lytro plenoptic camera that simultaneously captures images at up to 12 focal planes. When accommodation at any given plane was simulated, the corresponding defocus blur at other depth planes was extracted from the stack of focal plane images. Depth information from pictorial cues, relative blur and stereoscopic disparity was separately introduced into the images. In 2AFC tasks, observers were required to indicate which of two patches extracted from these images was farther. Depth discrimination sensitivity was highest when geometric and stereoscopic disparity cues were both present. Blur cues impaired sensitivity by reducing the contrast of geometric information at high spatial frequencies. While simulated generic blur may not assist depth perception, it remains possible that dioptric blur from the optics of an observer’s own eyes may be used to recover depth information on an individual basis. The implications of our findings for virtual reality rendering technology are discussed.

## Introduction

Many vertebrate and invertebrate visual systems have evolved multiple mechanisms that, in principle, allow them to estimate depth information from natural scenes. Organisms with overlapping binocular visual fields can use stereoscopic disparity to reliably estimate depth. Organisms with a narrow depth of field and adequate spatial resolution can use differences in optical blur between image regions to estimate their relative depths, although the direction of depth difference requires the ability estimate differences in the phase as well as amplitude spectrum of formed images. More broadly, visually cognitive organisms that can employ top-down processing and memory, may encode static pictorial cues, dynamic optic flow cues and use knowledge about the relative sizes of identified objects to estimate their relative depths.

The human visual system is potentially able to exploit all of these sources of information. A great deal of research has examined the integration of depth cues with impoverished laboratory stimuli. Blakemore [1] was the first to extensively study the range of depth discrimination due to disparity cues alone. He used vertical slit shaped targets to map depth discrimination thresholds across the visual field, showing that disparity is a reliable cue to depth over a vast region of the visual field, and that stereo-acuity is maximal at the empirical horopter (i.e. the locus of points that appear to be fused binocularly). Rogers & Bradshaw [2] used sinusoidal corrugations defined by horizontal disparities to show that differential vertical perspective strongly affects the amount of perceived depth in stereoscopic images. Bülthoff & Mallot [3] employed flat and smooth-shaded ellipsoids in conjunction with a stereoscopic depth probe to show that reliable depth information can be recovered by combining shading and disparity cues. However, the relative contribution of these alternative cues to our unified sense of depth is not well understood under natural conditions.

It has been demonstrated that blur is a reliable cue to depth even near fixation, although its effective usefulness is strongly debated [4–11]. Both Marshall et al. [4] and Mather [5] showed that when other cues to depth are removed, occlusion edge blur (i.e. the amount of blur at the borders between blurred and sharp image regions) can elicit strong and consistent depth ordering effects. With regards to simulated blur, Marshall et al. also showed that some observers tend to perceive blurred textures as near when they are isolated from context. Mather and Smith [6] have however shown that the visual system does not appear to integrate depth cues from blur and disparity, primarily employing the disparity cue over the blur cue when both are available. Nefs [8] has shown that manipulating the depth of field in photographs of semi-natural scenes alters perceived depth/width ratios. Held et al. [9] recently employed a volumetric stereo display and found that away from fixation, blur is a more precise cue to depth than disparity, and that when both cues are available, the visual system relies on the more informative cue. In the experiments conducted by Held et al. [9] and Hoffman & Banks [7], blur only ever increased with depth and the target with greater blur was always at a greater depth. However, in real environments, blur increases with distance in either direction away from the accommodation plane. Thus, a more blurred target could either be closer to or further from the observer than the object at his/her fixation plane. Indeed Vishwanath [10] criticized the Held at al. study by pointing out that what was effectively measured were blur discrimination thresholds, not perceived depth from blur. Langer and Siciliano [11] have attempted to replicate the findings from Held et al. employing conventional display technology, but found that with simulated blur subjects were unsuccessful at employing blur to discriminate depth at distances far from fixation. It is also worth noting that stereoscopic acuity covaries with spatial frequency [12–14], and since blur removes high spatial frequencies, blur could potentially impair depth perception.

Apart from notable exceptions [15–17], the experiments performed on blur and disparity have generally used impoverished synthetic stimuli, such as lines, gratings and random dot stereograms. These stimuli intentionally avoid monocular cues such as geometric perspective, which are present in everyday viewing of natural scenes. However, it has been suggested that the perception of visual space is determined by priors based on the probability distribution of real-world sources of retinal images [18]. We therefore examined depth cue combination with more complex naturalistic stimuli in which we were able to independently manipulate alternative sources of depth information. In particular, the experiments described in this work tested the relative contribution of spatial structure (relative size and geometry) due to perspective, binocular disparity, and defocus blur to depth perception using both a temporal and a spatial two-alternative forced choice (2AFC) paradigm. By comparing depth discrimination with and without both defocus blur and disparity in naturalistic images, we aimed to assess the role of each of these simulated depth cues in naturalistic, yet virtual, viewing conditions.

Understanding the interactions between these cues is particularly important when dealing with computer generated stereoscopic three dimensional scenes. In virtual reality environments for example, observers can experience visual fatigue, nausea and diplopia when depth cues are not accurately simulated [19–22]. We have previously shown, with the same technology employed in the current study, that the distribution of blur arising from light field photographs of natural images can be employed to facilitate binocular fusion and modify eye movement behavior [23]. Here we examine whether the same kind of rendered blur can successfully be employed to enhance depth perception in virtual reality applications. Disparity, whether it arises from natural viewing or artificially rendered through display technology, is a remarkably robust cue to depth. Blur arising from the optics of an individual’s eye might also be a reliable cue to depth, but replicating an individual’s optical aberrations in display rendering technology is currently feasible only with adaptive optics methods [24]. Thus assessing whether depth perception from rendered blur is also robust in naturalistic virtual reality may be useful towards bettering current virtual reality technology.

## Materials and Methods

### Subjects

Four subjects completed the first experiment, one of whom (GM) was an author of this work. Four subjects completed the second experiment, one was an author (GM) and one had participated in the first experiment. All subjects reported normal or corrected to normal acuity and normal stereo vision. All procedures adhered to the tenets of the declaration of Helsinki and were approved by the Northeastern University Institutional Review Board. All subjects provided written informed consent.

### Apparatus

The experiments were created using Psychophysics Toolbox Version 3 [25, 26] with Matlab version R2011a (Mathworks). Stimuli were presented on a Samsung SynchMaster 2233 LCD monitor with a resolution of 1680x1050 pixels at 120 Hz, run from an NVidia Quadro FX580 graphics processing unit. Observers were seated 50cm in front of the monitor with their heads set in a chin rest. Display dot pitch was 0.282 mm and the monitor subtended 49x33 degrees. Stereoscopic disparity was presented via the NVIDIA 3D Vision kit. The cross talk of the system was 1% measured with a Spectrascan 6500 photometer using the methods described by [27].

### Stimuli

**Fig 1:**
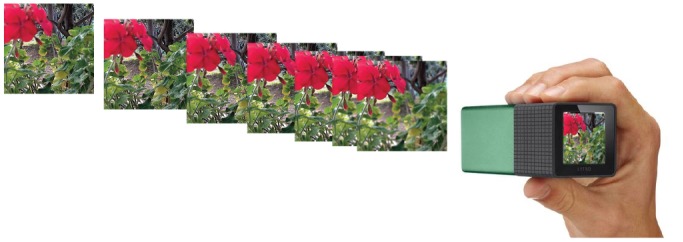
Lytro plenoptic camera. Each photographic exposure generated a stack of up to 12 images, each in focus at a different depth plane.

The stimuli for the psychophysical experiments were extracted from light field photographs of natural scenes taken with Lytro plenoptic camera (Lytro Inc, CA). The Lytro is a light field camera that simultaneously captures several versions of the same image, each with a different focus. This property allowed us to vary the level of defocus blur across the image in proportion to the distance in depth from the plane of focus (Fig 1).

A software tool was used to extract data and *.jpg* files from the Lytro file format [28]. For each photograph, a.jpg image was generated at each focal plane, a depth map of the scene, a look up table that specifies the depth at which each image in the stack is in focus, and the camera settings (including exposure, aperture, focal length, ISO) for each photograph. The image stack produced by the Lytro contained between 1 and 12 images, depending on the depth structure of the scene. If the scene contained many objects at depths near to the camera, more images were output. For panoramic scenes, where everything is effectively in focus at infinity, only one image was generated.

**Fig 2:**
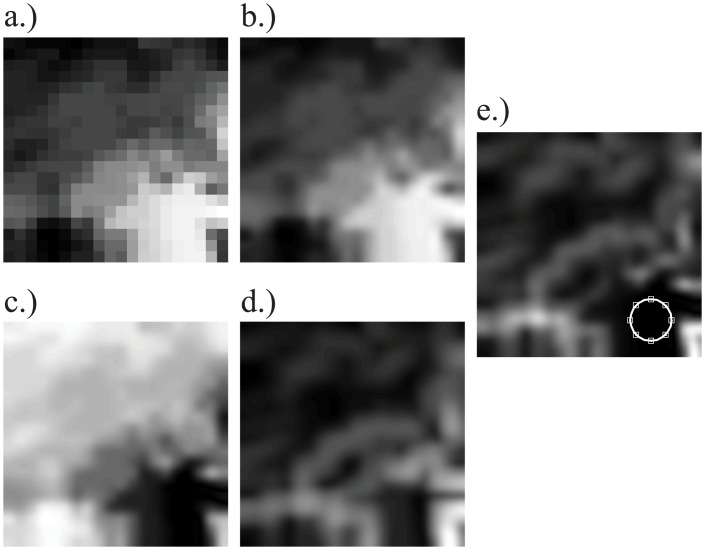
Steps to determine the in-focus area for the first (closest to the camera) image in the stack. a) The original 20x20 pixel depth map was (b) up-sampled to 1080 pixels and smoothed with a Gaussian, so that (c) the distance in depth of each pixel from the first image’s focal depth could be computed. d) The local standard deviation of the depth image, computed over a 165x165 pixel region, was used to identify areas of uniform depth. e) The product of c and d: the white circle is centered on the location where this product has the lowest value (closest depth and lowest variance), to indicate the position of the in-focus patch at this depth.

The photographer (author GM) positioned the camera at eye level at locations where the closest object was approximately 10 cm from the lens. Examples of representative stimuli are available at: https://pictures.lytro.com/guido_maiello. Ten light field photographs were selected by the author to include only those in which 11 or 12 focal plane images were generated. For each photograph, the depth map and the depth look up table were used to calculate the location corresponding to the focal depth of each image in the stack. This involved finding the pixel location in which each image of the stack had an in-focus patch. To do this, the first step was resizing the depth map, using bicubic interpolation, from its original size of 20x20 pixels (Fig 2a) to be the same size as the focal plane images (1080x1080 pixels). The obtained depth image was further smoothed with a 2D Gaussian low-pass filter of size 3x3 pixels and standard deviation of 0.5 pixels (Fig 2b).

**Fig 3:**
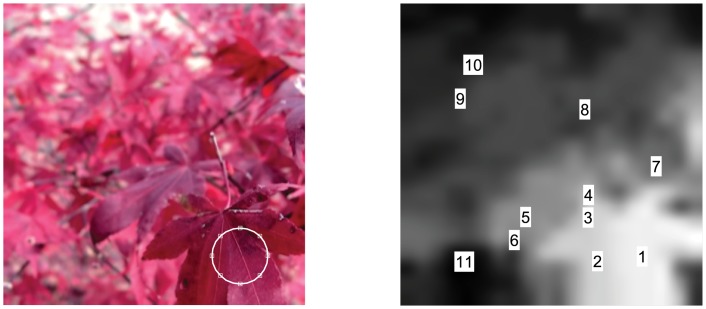
Example of extracted in-focus patches for each focal plane. Left: in-focus patch for the first image in the stack. Right: locations of in-focus patches on the depth map.

For each image in the stack, the corresponding focal depth value was retrieved from the depth look up table. The depth value corresponded to the focal depth at which the image was in-focus. The distance of this depth value from the depth values in the up-sampled and smoothed depth map was computed as a 1080 x 1080 matrix (Fig 2c). This matrix was then multiplied by the local standard deviation (computed over a 165x165 pixel region) of the up-sampled depth map in order to give greater weight to the parts of the image which had more homogeneous depth distributions [29], as shown in Fig 2d. The minimum of the product of the depth and depth variance matrix was taken to be the pixel coordinates in which the image was in-focus (Fig 2e). Fig 3 shows an example of the results of this method. The left image shows the in-focus patch identified for the first image of the stack. The right image shows the locations on the depth map where in-focus patches were found for all 11 images in the stack.

**Fig 4:**
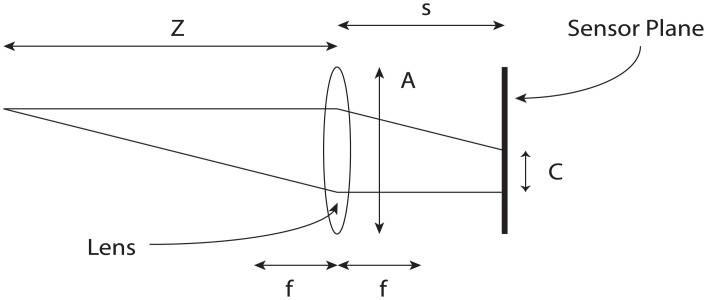
Blur in a conventional lens system. An object at distance Z from a lens with focal length f and aperture A is imaged on a sensor plane at distance s. The object will be blurred over a circular region with diameter C.

The Lytro camera captures light field pictures using a micro-lens array between the image sensor and the main lens. The technical details are extensively explained in [30]. In a conventional lens system, blur can be defined as the diameter of the circle *C* over which the point at distance *Z* is imaged on the image sensor placed at distance *s* from a lens with focal distance *f* and aperture *A* (Fig 4). *C* can be defined as [31]:

$$\begin{array}{c} C=As|\frac{1}{f}-\frac{1}{Z}| \end{array}$$ (1)

The Lytro software outputs depth information in lambda units. One lambda is the distance the sensor plane of a conventional camera must move to change the diameter of the circle of confusion *C* by the pitch of the micro-lens array. The pitch of the micro-lens array of the camera employed was specified to be 13.9 *μ*m, the focal distance was 6.5 mm, and the aperture was f/2. The circle of confusion when moving the sensor plane from distance *f* (focus at infinity) to *s* is given by:

$$\begin{array}{c} C=\frac{As}{Z} \end{array}$$ (2)

By using the thin lens equation and rearranging, object displacement in diopters can be expressed as

$$\begin{array}{c} d_{0}=\frac{1}{f}-\frac{1}{s} \end{array}$$ (3)

where the displaced focal plane s is given by:

$$\begin{array}{c} s=\frac{f(A+C)}{A} \end{array}$$ (4)

**Fig 5:**
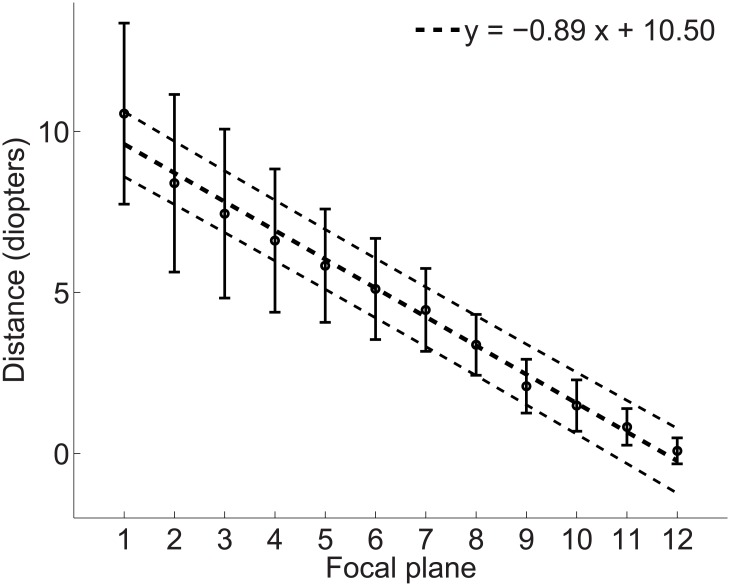
Dioptric depth separation between image planes for the ten light field pictures in the study. Black circles represent the mean dioptric distance for each image in the stack. Error bars are ±1 standard deviation. Dotted lines represent the linear fit (coarse dots) with 95% confidence intervals (fine dots).

Through these calculations it is possible to recover the dioptric distance at which each image in the stack was in focus. Fig 5 shows the mean distance for the images in the stack across all ten light field photographs in the study. The mean dioptric distance between two image planes was 0.89 diopters.

In the Experiments, a Reference and a Test image patch were selected from differing depth planes in the same scene. Reference and Test stimuli were presented in circular windows, with a diameter of 6° and whose edges were smoothed over 0.6° with a raised cosine envelope. Subjects judged the relative depth of Reference and Test patches. There were four conditions in which stimuli contained differing sources of depth information:

1. Pictorial: Reference and Test patches were in-focus with zero disparity. Observers could only employ naturally-occurring pictorial cues to perform depth ordering, since no depth information was present from disparity of defocus blur.
2. Pictorial plus Blur: in addition to any naturally-occurring pictorial cues, the Reference patch was in focus, to simulate accommodation at the plane of the reference image, while the Target patch was blurred proportionally to the depth separation between the reference and target patches. Both patches were taken from the same image in the Lytro stack, so that the patch that was in-focus simulated accommodation at that plane, and the patch that was out of focus simulated appropriate defocus blur for the relative depth of that plane. Image blur arose directly from the optics of the Lytro camera, and was not added via image processing.
3. Pictorial plus Disparity: in addition to any naturally-occurring pictorial cues, Reference and Target patches were offset with appropriate disparity proportional to the depth separation between the patches. Both stimuli were in-focus. The Reference patch was presented at zero disparity, the Target disparity was crossed or uncrossed according to the distance in depth between the patches. The absolute values of the disparities ranged from 4 to 40 arcmin.
4. Pictorial plus Blur and Disparity: pictorial, blur and disparity depth cues were all present and coherent. The Reference patch was presented in focus at zero disparity, the blur and disparity of the Target patch were proportional to the separation in depth between the patches.

### Procedure

#### Temporal 2AFC Procedure

**Fig 6:**
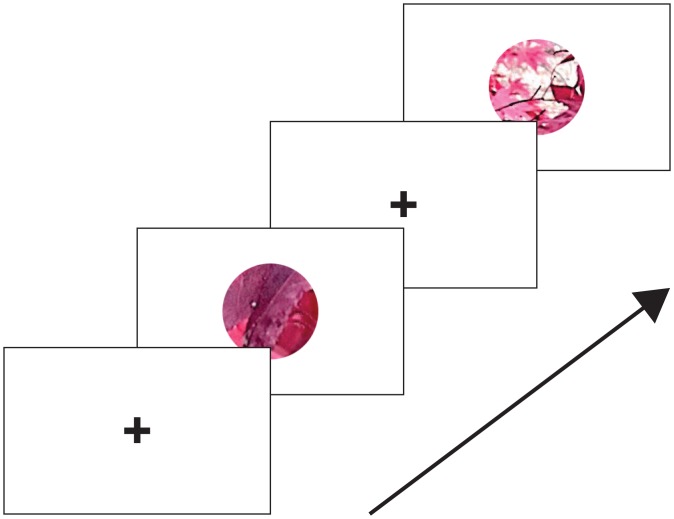
Example of one trial from Experiment 1. Each trial began with a blue fixation cross presented in the center of a grey screen for 1 s. Two stimuli were presented in a circular window with abrupt onset and offset for 200 ms, separated by a 500 ms interval containing only the fixation cross. The subject was required to fixate the cross and to indicate whether the first or second patch was farther, using any available cues.

The sequence of events from a typical trial is shown in Fig 6. Each trial started with the presentation of a central fixation cross on a grey background. After 1 second, two circular image patches were shown for 200 ms, separated by a 500 ms inter-stimulus interval in which the fixation cross was again displayed. Observers were required to indicate by pressing one of 2 buttons, which of the two image patches presented in the two intervals was further. Feedback was given by a green fixation cross flash for a correct answer or red for an incorrect answer.

There were 10 repetitions for each of the 10 depth pairs for each of the four conditions. Thus each session consisted of 400 trials, 100 trials per depth cue condition. Each subject completed each session twice, for a total of 800 trials per subject. The presentation order of the Reference and Test stimulus and the order of the presentation of the conditions was random.

#### Spatial 2AFC Procedure

In the temporal 2AFC procedure, the visual targets containing blur and disparity appeared at fixation and might have induced vergence and possibly accommodation away from the fixation plane. Additionally, presenting blur and disparity at the center of the visual field is not typical for real world conditions where binocular fixation projects focused images with zero disparity on the fovea. Objects nearer or farther than the plane of fixation will instead be projected onto the peripheral visual field, at different retinal disparities, and will be out of focus. We therefore repeated Experiment 1 except with a spatial paradigm to keep vergence and accommodation at the plane of fixation (the surface of the computer monitor), and simultaneously presented cues at other depth planes. The paradigm was the same as in Experiment 1, except that the reference and target patches were presented concurrently at 4 degrees to the left and right of fixation.

**Fig 7:**
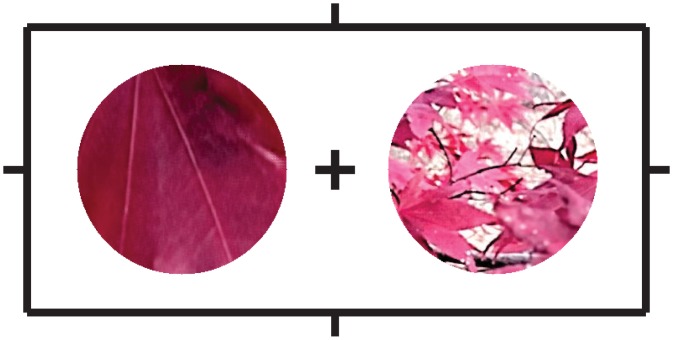
Example of one trial from Experiment 2. While fixating on the central cross, a subject was required to indicate which patch, the left or the right, was farther, using any available cues.

Each trial started with the presentation of a blue central fixation cross and white peripheral nonius lines on a grey background. The nonius lines served to aid vergence on the surface of the monitor. After 1 s, two circular image patches were shown for 200 ms (Fig 7). Subjects were instructed to fixate on the central cross and report, by pressing one of two computer keys, whether the left or the right image patch appeared farther. The fixation cross was green following a correct response or red following an incorrect response.

### Analysis

To measure discrimination sensitivity the data for each subject and condition were converted into discrimination d’ [32]. To compute group confidence intervals on d’ measurements a bootstrapping procedure was employed [33]. Mean d’ for each observer and each condition were computed from the original data resampled with replacement 5000 times. These bootstrapped distributions were then collapsed across observers to obtain group distributions for each condition. The group distributions were fit with a Gaussian function from which the 2.5th and 97.5th quantiles were taken as 95% confidence intervals. Mean and standard deviation of fitted group distributions were employed to Z-transform group means and compute p-values. Group averages for different conditions that fell outside the bounds of each other’s confidence intervals (corresponding to p-values < 0.05) were considered to be significantly different from one another.

## Results

### Experiment 1: Temporal 2AFC Procedure

**Fig 8:**
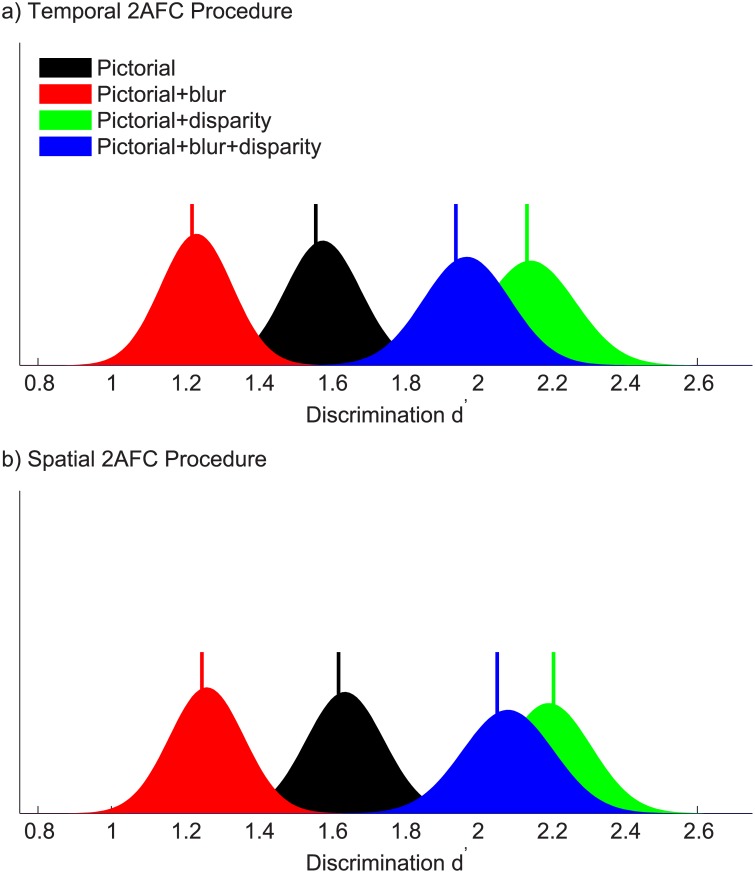
Discrimination sensitivity. Discrimination sensitivity d’ for each condition in the (a) temporal 2AFC task and (b) spatial 2AFC task, averaged over the four observers in each experiment. The graphs show group bootstrapped distributions of discrimination d’ for the four experimental conditions. Vertical bars represent the true group means.

Fig 8a shows discrimination d’ for the four experimental conditions averaged across observers. When only pictorial cues to depth were present, discrimination was already possible (d’ = 1.56, 95% confidence intervals [1.39 1.78]). When blur cues were introduced, discrimination significantly worsened (d’ = 1.22, 95% confidence intervals [1.04 1.43], p = 10^−3^). When disparity cues were introduced alongside pictorial cues, discrimination improved significantly (d’ = 2.14, 95% confidence intervals [1.91 2.40], p = 10^−7^). When both blur and disparity cues were present alongside pictorial cues, discrimination was again significantly better than with pictorial cues alone (d’ = 1.94, 95% confidence intervals [1.75 2.23], p = 10^−3^), and not significantly different from the pictorial + disparity condition (p = 0.11).

As expected, depth order discrimination was already possible solely with pictorial cues to depth. Binocular disparity, which is a robust cue to depth perception, significantly improved discrimination sensitivity. Contrary to expectation, the addition of blur cues to depth impaired depth order discrimination, probably by reducing the contrast of pictorial depth cues at high spatial frequencies. However, blur did not significantly impact depth order discrimination due to binocular disparity, suggesting that disparity is a robust cue to depth even with low spatial frequency information.

### Experiment 2: Spatial 2AFC Procedure

Fig 8b shows discrimination d’ for the four experimental conditions averaged across observers. When only pictorial cues to depth were present, discrimination was possible (d’ = 1.62, 95% confidence intervals [1.43 1.85]), confirming the results of Experiment 1. When blur cues were introduced, discrimination significantly worsened (d’ = 1.25, 95% confidence intervals [1.07 1.46], p = 10^−4^). When disparity cues were introduced alongside pictorial cues, discrimination improved significantly (d’ = 2.21, 95% confidence intervals [1.96 2.43], p = 10^−7^). When both blur and disparity cues were present alongside pictorial cues, discrimination was again significantly better than with pictorial cues alone (d’ = 2.05, 95% confidence intervals [1.84 2.34], p = 10^−4^), and not significantly different from the pictorial + disparity condition (p = 0.19).

For both experiments, we repeated the analysis using conventional detection thresholds, fit with logistic psychometric functions (S1 Fig and S2 Fig), and found a similar pattern of results (*r* ^2^ = 0.86, *p* = 10^−6^ for Experiment 1; *r* ^2^ = 0.90, *p* = 10^−7^ for Experiment 2). We however choose to present the d’ analyses because it provides a more appropriate comparison of discrimination performance between experimental conditions.

## Discussion

For binocular organisms with well-developed visual cognition, multiple cues are available in natural environments to indicate the absolute or relative depth of different objects. While combinations of these alternate cues have been studied with simplified laboratory stimuli, there have been relatively few attempts to examine depth cue combination in natural scenes. We used a plenoptic camera to capture light field images of real scenes, which allowed us to control and combine depth cues from pictorial, binocular disparity and defocus blur sources. We found that even when pictorial information was impoverished by a small field of view, partial occlusion and parafoveal viewing, pictorial cues alone enabled relative depth judgments. Stereoscopic disparity provided significant benefit for depth perception both in the near periphery and at the fovea. Contrary to recent data [9], we find that the addition of defocus blur cues to depth impaired depth perception.

Landy et al. [34] propose a model of depth cue combination termed modified weak fusion (MWF), which consists of a dynamically weighed average of available cues, based upon the estimated reliability of the cues. In our study we found that depth order discrimination was already possible with pictorial cues alone. This supports previous evidence that pictorial cues such as texture perspective are strong cues to depth that can be used in combination with other depth cues and can at times take precedence over other depth cues such as motion parallax [35, 36].

Held et el. have recently shown that depth discrimination thresholds for random dot patterns decrease when blur is varied in proportion to depth [9]. We found instead that adding dioptric defocus blur cues to depth actually impaired depth perception. The main difference between our study and the previous is that Held et al. employed a volumetric display to present observers with blur due to the optics of their own eyes. Thus, in the Held study, observers might have been able to employ other optical cues, such as chromatic aberration, that have been shown to be possible cues to depth sign [37]. Furthermore, in the study by Held et al., an increase in blur was always correlated with an increase in depth away from fixation, thus the observers could perform the task using blur discrimination (the decrease in depth was pilot tested but the data were not presented). In the present study, blur increased with distance away from the plane of focus, regardless of the sign of the distance (i.e. closer or further).

The addition of blur cues impaired depth discrimination by reducing the contrast of geometric and disparity information at high spatial frequencies. According to previous work, subjects should have been able to perform depth ordering using image defocus blur as a cue to depth [9, 37] by solving the sign problem using pictorial cues. Although the amplitude of contrast attenuation from defocus blur is an unsigned quantity, relative phase differences between near and far objects of equal blur could, in principle, be used to estimate the sign of depth difference. Our results instead suggest that subjects were unable to use generic defocus blur in the optics of the Lytro camera as a cue to depth.

The results from the spatial 2AFC experiment confirmed the results from the temporal 2AFC paradigm. Accommodative response to blur did not seem to influence the results, possibly because accommodative latency is greater (300 ms) [38] than the stimulus duration we employed (200 ms). Fusional vergence during the 2AFC temporal experiment could have affected the stimulus, since the typical vergence onset latency (approximately 160–180 ms) [39] is less than the stimulus duration we employed. In the temporal 2AFC paradigm we would have expected improved performance in the stereoscopic conditions by added depth information from oculomotor vergence cues with respect to the spatial experiment. The fact that we did not observe differences between the paradigms supports previous evidence that stereopsis is a direct process [40], where depth is estimated from retinal disparity and not from fusional vergence responses.

Since the defocus blur depth cue did not arise from the optics of an individual’s eye, it is possible that subjects could learn the correct association between defocus blur of the Lytro camera and object depth, in the same way that they may have learned this relationship for their own optics. In our study, feedback was given to verify whether subjects could learn that association. We found no evidence of perceptual learning in either the temporal nor the spatial experiment (S3 Fig).

To estimate depth discrimination sensitivity, data were pooled over all depth differences. Because sensitivity to different cues may change with depth difference, it remained possible that blur might have had an adverse effect at some depth differences, and a positive effect on depth discrimination at others. However, we found no evidence of an association between sensitivity to the different cues and the magnitude of the depth differences (S4 Fig). Furthermore, in all cases, sensitivity decreased with the addition of blur (compare S4A and S4B; or S4C and S4D Fig).

Furthermore, we considered the possibility that blur may have had different effects at far or near absolute distances. To address this question, we fit raw discrimination data with asymmetric logistic functions (S1 Fig and S2 Fig) and compared the symmetry of the fitted functions. We found no evidence that blur asymmetrically modified depth discrimination do to perspective (symmetry of pictorial vs symmetry of pictorial+blur conditions: p = 0.12 for Experiment 1; p = 0.26 for Experiment 2, paired samples t-test) or disparity cues (symmetry of pictorial+disparity vs symmetry of pictorial+disparity+blur conditions: p = 0.31 for Experiment 1; p = 0.19 for Experiment 2, paired samples t-test). This suggests that pooling our data did not mask conditions in which blur is beneficial. Instead, the results indicate that in naturalistic images with signed depth, image blur does not facilitate depth discrimination.

These observations confirm our conclusion that generic defocus blur is unlikely to be successful at facilitating depth perception in naturalistic images. This result has important implications for virtual reality applications. We propose that for virtual reality applications, the addition of dioptric blur, which can provide usable cues to depth in laboratory settings [4–7, 9, 10, 37], is too unreliable when presented with other, more reliable, depth cues. Consequently, generic defocus blur may be negligibly weighted by the visual system in the recovery of depth information in naturalistic images that contain multiple potential depth cues. This conjecture is supported by the observation that depth discrimination was best when geometric and stereoscopic disparity cues were both present. This indicates that these cues can be combined, as previously reported by [41] and [34] who have proposed weighted cue combination rules. When [41] introduced blur cues along with geometric and disparity cues, they marginally impaired discrimination, in agreement with our observations and suggesting that disparity and pictorial cues are weighed stronger than defocus blur. Furthermore this result is in line with the notion that disparity can be used even with low spatial frequency information [42] that is encoded by coarse disparity detectors [12, 43] and still produce stereoscopic depth percepts.

The main implication of our findings with regards to virtual reality technology is that rendered generic defocus blur will not necessarily facilitate the fine perception of relative depth in complex 3D stimuli. Furthermore, applying blur to pictorial depth cues may even reduce their effectiveness, especially when information is carried by high spatial frequencies, and this detrimental effect might hinder depth cue combination. Nevertheless, blur might play other roles in virtual reality rendering techniques, as other studies have found that rendered blur has an array of effects on the perceptual experience of virtual reality. We have recently shown that rendering blur can produce benefits on binocular fusion of stereoscopically rendered naturalistic scenes [23] and may thus improve visual comfort in virtual reality applications. Vishwanath & Blaser [44] have shown that blur gradients, which can be presented as independent from the depth structure of the rendered scene, can modulate the perception egocentric distance, and play a role in the tilt-shift miniaturization effect. Similarly, Wang et al. [15] provided evidence that a globally blurred background enhances the perceived depth separation between the background and a sharp, disparity defined foreground object. Nefs [8] found that depth of field systematically affected perceived depth/width ratio of photographs of natural scenes, while Zhang et al. [17] have shown complex patterns of dependencies between depth of field, height-in-the-field, 3D display system, and perceived depth when observers were asked to draw floor plans of viewed scenes to scale. All these studies find that blur modulates the perception of global scene depth. Thus blur might still be gainfully employed in virtual reality applications to modulate egocentric distance and global scene appearance (much like what is done in cinematography [45, 46]), and to potentially facilitate oculomotor behavior.

## Supporting Information

S1 Fig Psychometric functions of depth discrimination as a function of depth plane separation for the four experimental conditions (columns) and four subjects (rows) for Experiment 1. Data points show percent ‘target farther’, error bars show binomial standard deviation. Solid line shows the best fitting asymmetric logistic function along with 68% confidence intervals (dashed lines).
(EPS)

S2 Fig Psychometric functions of depth discrimination as a function of depth plane separation for the four experimental conditions (columns) and four subjects (rows) for Experiment 2. Data points show percent ‘target farther’, error bars show binomial standard deviation. Solid line shows the best fitting asymmetric logistic function along with 68% confidence intervals (dashed lines).
(EPS)

S3 Fig Percent change in performance as a function of trial number for the (a) temporal and (b) spatial 2AFC tasks averaged over the four observers. The graphs show average percent change in performance for the four experimental conditions. Error bars represent ±1 standard deviation. Across subjects and conditions we observe only random variations around baseline performance, with no evidence of learning effects.
(EPS)

S4 Fig Discrimination d’ plotted against depth plane separation for the four experimental conditions. (a) Pictorial cue (b) Pictorial+blur cues (c) Pictorial+disparity cues (d) Pictorial+blur+disparity cues. Discrimination d’ for each level of depth plane separation (asterisks) is computed from discrimination data aggregated across observers and experiments. Filled lines are best fitting linear equations passing through the data, bounded by 68% confidence intervals of the fit (shaded regions).
(EPS)
